# Scalable symmetry detector and its applications by using beam splitters and weak nonlinearities

**DOI:** 10.1038/s41598-017-15691-0

**Published:** 2017-11-10

**Authors:** Ying-Qiu He, Dong Ding, Feng-Li Yan, Ting Gao

**Affiliations:** 1 Department of Biomedical Engineering, Chengde Medical University, Chengde, 067000 China; 2 Department of Basic Curriculum, North China Institute of Science and Technology, Beijing, 101601 China; 3 College of Physics Science and Information Engineering, Hebei Normal University, Shijiazhuang, 050024 China; 4 College of Mathematics and Information Science, Hebei Normal University, Shijiazhuang, 050024 China

## Abstract

We describe a method to detect twin-beam multiphoton entanglement based on a beam splitter and weak nonlinearities. For the twin-beam four-photon entanglement, we explore a symmetry detector. It works not only for collecting two-pair entangled states directly from the spontaneous parametric down-conversion process, but also for generating them by cascading these symmetry detectors. Surprisingly, by calculating the iterative coefficient and the success probability we show that with a few iterations the desired two-pair can be obtained from a class of four-photon entangled states. We then generalize the symmetry detector to *n*-pair emissions and show that it is capable of determining the number of the pairs emitted indistinguishably from the spontaneous parametric down-conversion source, which may contribute to explore multipair entanglement with a large number of photons.

## Introduction

Since optical quantum systems provide some natural advantages, they are prominent candidates for quantum information processing^1–3^. As a fundamental physical resource, the multiphoton entanglement plays a crucial role in optical quantum computing^4–6^. A standard entangled photon pair is created by means of the nonlinear optical process of spontaneous parametric down-conversion (SPDC)^7^. In SPDC process, one may create photons entangled in various degrees of freedom, for example, polarization entanglement^8–10^, path entanglement^11,12^, etc.

For creation of polarization-entangled photons, a simplified Hamiltonian^13,14^ of the nonlinear interaction is given by ${H}_{{\rm{SPDC}}}={\rm{i}}\kappa ({\hat{a}}_{H}^{\dagger }{\hat{b}}_{V}^{\dagger }-{\hat{a}}_{V}^{\dagger }{\hat{b}}_{H}^{\dagger })+{\rm{H}}{\rm{.c}}{\rm{.}}$, where ${\hat{a}}_{x}^{\dagger }$ and ${\hat{b}}_{x}^{\dagger }$ (with *x* = *H*, *V*) are respectively the creation operators with horizontal (*H*) or vertical (*V*) polarization in the spatial modes *a* and *b*, and *κ* is a real-valued coupling constant depended on the nonlinearity of the crystal and the intensity of the pump pulse. In the number state representation, the resulting photon state reads^14–16^

1 $$|{\rm{\Psi }}\rangle =\frac{1}{{\cosh }^{2}\tau }\sum _{n=0}^{\infty }\,\sqrt{n+1}{\tanh }^{n}\tau |{\psi }_{n}^{-}\rangle ,$$

2 $$|{\psi }_{n}^{-}\rangle =\frac{1}{\sqrt{n+1}}\sum _{m=0}^{n}\,{(-\mathrm{1)}}^{m}|n-m{\rangle }_{{a}_{H}}|m{\rangle }_{{a}_{V}}|m{\rangle }_{{b}_{H}}|n-m{\rangle }_{{b}_{V}},$$

where e.g. $|m{\rangle }_{{a}_{V}}$ means *m* vertically polarized photons in spatial mode *a*, and *τ* = *κt/ħ* is the interaction parameter with *t* being interaction time. Each $|{\psi }_{n}^{-}\rangle$ represents the state of *n* indistinguishable photon pairs with 〈*n*〉 = 2sinh^2^ *τ*. It should be noted that $|{\psi }_{n}^{-}\rangle$ is different from the general multiphoton entangled state in which each photon represents a qubit. State (2) is usually called the twin-beam multiphoton entangled state.

To avoid multipair emission events, in general, *τ* is restricted to small enough, such that mainly the first-order term has been taken into account. For the higher-order terms, these twin-beam multiphoton entangled states have interesting features^15^, i.e. they are not only entangled in photon number for the spatial modes *a* and *b*, but also entangled maximally in polarization degree of freedom. Especially for the second-order emission, it has been shown that^17^, depending on the relation between the duration of the pump pulse and the coherence time of the photons, the emitted state is described by two independent pairs or an indistinguishable twin-beam four-photon entangled state. For the indistinguishable four-photon entangled state, furthermore, it can be useful for the applications in quantum information processing, for example, testing the quantum formalism against the local realistic theories^18,19^. Unfortunately, up to now there are only a few reports^20–22^ to exploring these analog of a singlet state of two spin-*n*/2 particles.

In this paper, we first focus on the twin-beam four-photon entangled states and design a quantum circuit of symmetry detector to evolve them by using a beam splitter (BS) and weak nonlinearities. By cascading symmetry detectors, we then propose a scheme of generating the twin-beam two-pair entangled state in a near deterministic way. Finally, we generalize the present symmetry detector to high-order emissions.

## Results

### Symmetry detector based on beam splitter and weak nonlinearities

Throughout the text, for simplicity we write |*m*, *n*; *r*, *s*〉 as an abbreviation for state $|m{\rangle }_{{a}_{H}}\otimes |n{\rangle }_{{a}_{V}}\otimes |r{\rangle }_{{b}_{H}}\otimes |s{\rangle }_{{b}_{V}}$ which means that there are *m* horizontally polarized photons and *n* vertically polarized photons in spatial mode *a* and also there are *r* horizontally and *s* vertically polarized photons in spatial mode *b*.

We first restrict our attention to the four-photon entanglement and describe a method to explore symmetry detector for the twin-beam entangled states. In general, consider a class of four-photon entangled states

3 $$|{\rm{\Phi }}\rangle =N\mathrm{(|2},0;0,2\rangle +\mathrm{|0},2;2,0\rangle -c\mathrm{|1},1;1,1\rangle ),$$

where *c* is a constant and normalization factor *N* satisfies *N* ^2^ = 1/(2 + |*c*|^2^). Without loss of generality, we may suppose coefficient *c* to be real.

**Figure 1:**
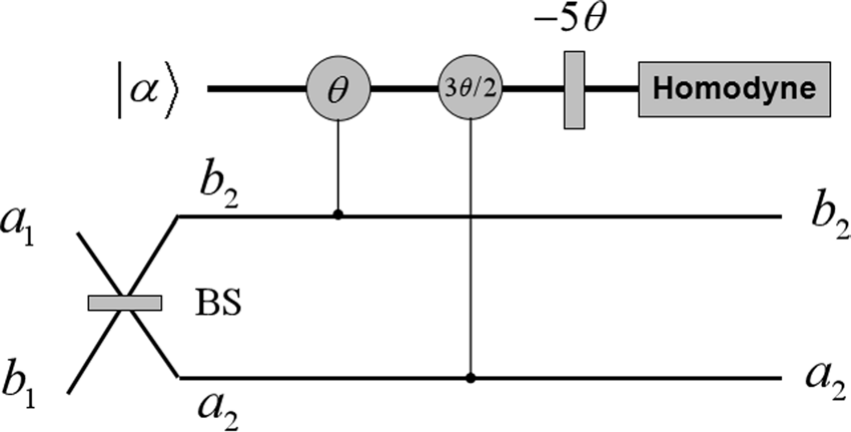
The schematic diagram of symmetry detector based on a beam splitter (BS) and weak nonlinearities. *a*_1_, *b*_1_ are input ports of a 50:50 BS, and *a*_2_, *b*_2_ are the corresponding outputs, respectively. |*α*〉 is a coherent state in probe mode. *θ* and 3*θ*/2 are phase shifts on the coherent probe beam due to the interaction between photons in signal and probe modes. −5*θ* is a single phase gate.

Consider a lossless 50:50 BS with Hamiltonian ${H}_{{\rm{BS}}}=-{\rm{i}}\pi ({\hat{a}}^{\dagger }\hat{b}-\hat{a}{\hat{b}}^{\dagger })/4$, where ${\hat{a}}^{\dagger }$ (${\hat{b}}^{\dagger }$) and $\hat{a}$ ($\hat{b}$) are respectively creation and annihilation operators in the input spatial mode *a* (*b*). As shown in Fig. 1, since the interference effect of BS, the input twin-beam state evolves

4 $$|{{\rm{\Phi }}}_{{\rm{BS}}}\rangle =N^{\prime} \mathrm{[(1}-c\mathrm{)(|2},2;0,0\rangle +|0,0;2,2\rangle )+\mathrm{(1}+c)(\mathrm{|2},0;0,2\rangle +|0,2;2,0\rangle )-2|1,1;1,1\rangle ],$$

where (*N*′)^2^ = 1/(4*c* ^2^ + 8). An interesting consequence of this evolution is that the input state yields two possible cases, i.e. symmetric state (two photons are in one spatial mode and others are in another spatial mode) and alternatively asymmetric state (the output four photons are in the same spatial mode).

In order to distinguish between the symmetric state and the asymmetric state, we here consider quantum nondemolition detection^23,24^ by using weak nonlinearities. As an important nonlinear component for all-optical quantum computing, Kerr medium^25–27^ is capable of evolving photons in signal and probe modes with the interaction Hamiltonian ${H}_{{\rm{Kerr}}}=\hslash \chi {\hat{n}}_{s}{\hat{n}}_{p}$, where *χ* is the coupling strength of the nonlinearity and ${\hat{n}}_{s}$ (${\hat{n}}_{p}$) represents the number operator for the signal (probe) mode. As a result, if there are *n* photons in the signal mode, then it yields *nθ* in the probe mode, where *θ* = *χt* is a phase shift on the coherent probe beam induced by the interaction via Kerr media and *t* represents the interaction time. In this way, these Kerr media are used mainly in creating and manipulating multiphoton entanglement^28–42^. Since the Kerr nonlinearities are extremely weak^27,31^, we here only take small but available phase shifts into account.

As shown in Fig. 1, after an overall interaction between the photons with Kerr media, the combined system $|{{\rm{\Phi }}}_{{\rm{BS}}}\rangle \otimes |\alpha \rangle$ then evolves as

5 $$\begin{array}{c}|{{\rm{\Phi }}}_{{\rm{CK}}}\rangle =\sqrt{1-P}\mathrm{(|2},2;0,0\rangle |\alpha {{\rm{e}}}^{{\rm{i}}\theta }\rangle +\mathrm{|0},0;2,2\rangle |\alpha {{\rm{e}}}^{-{\rm{i}}\theta }\rangle )/\sqrt{2}+\sqrt{P}{N}_{1}\mathrm{(|2},0;0,2\rangle \\ \quad \quad \quad \,+\mathrm{|0},2;2,0\rangle -{c}_{1}\mathrm{|1},1;1,1\rangle )|\alpha \rangle ,\end{array}$$

where *c*_1_ = 2/(1 + *c*) is the derived coefficient connected with the original *c*, *P* = 1/{1 + (1 − *c*)^2^/[2 + (1 + *c*)^2^]} and ${N}_{1}^{2}=1/\mathrm{(2}+{c}_{1}^{2})$ are respectively the probability and normalization factor for the symmetric state.

We next turn to the question of how to project the signal photons into the symmetric state or the asymmetric state. For a real coherent state, generally, one may perform an *X* homodyne measurement^43–46^ with the quadrature operator $\hat{x}=\hat{a}+{\hat{a}}^{\dagger }$. In terms of the result^47^ 〈*x*|*α*〉 = (2*π*)^−1/4^ exp[−(Im(*α*))^2^ − (*x* − 2*α*)^2^/4], after the *X* homodyne measurement on the probe beam, for *x* > *α*(1 + cos *θ*), one can obtain the symmetric state

6 $$|{{\rm{\Phi }}}_{x}^{1}\rangle ={N}_{1}\mathrm{(|2},0;0,2\rangle +\mathrm{|0},2;2,0\rangle -{c}_{1}\mathrm{|1},1;1,1\rangle ).$$

Alternatively, for *x* < *α*(1 + cos*θ*), we get the asymmetric state

7 $$|{{\rm{\Phi }}}_{x}^{0}\rangle =\mathrm{(|2},2;0,0\rangle +\mathrm{|0},0;2,2\rangle )/\sqrt{2},$$

up to a phase shift *φ*_*x*_ = −*α* sin *θ*(*x* − 2*α* cos *θ*)/2 mod 2*π* on the spatial mode *b*_2_ according to the value of the measurement.

That is, if one permits the phase shift with feedback from the value of the measurement, then the twin-beam four-photon asymmetric state $|{{\rm{\Phi }}}_{x}^{0}\rangle$ can be prepared. Also, for each symmetric state, it is interesting to note that the output state is analogous to the input state, up to a correlation coefficient (relative amplitude). Especially, for *c* = 1, i.e.

8 $$|{\rm{\Phi }}\rangle =\mathrm{(|2},0;0,2\rangle +\mathrm{|0},2;2,0\rangle -\mathrm{|1},1;1,1\rangle )/\sqrt{3},$$

it is the twin-beam entangled state emitted by the SPDC source with the duration of the pump pulse is much shorter than the coherence time of the photons, and we here refer to this pair of the indistinguishable four-photon entangled states as *two-pair*, for simplicity. Obviously, for this two-pair, one can immediately obtain the result that the output state is the same as the input.

### Two-pair generation by cascading symmetry detectors

For a four-photon entangled state created in SPDC process^17^, besides the above two-pair, the four photons can be emitted as two independent pairs in the opposite limit, or any intermediate situation depended on the ratio between the duration of the pump pulse and the coherence time of the created photons. Since the relative phase relation and the equal weight of the terms, as a singlet spin-1 state, the two-pair satisfies rotational symmetry. Similar to the rotationally symmetric Bell state, it may be useful for quantum information processing and quantum computation in the future.

**Figure 2:**
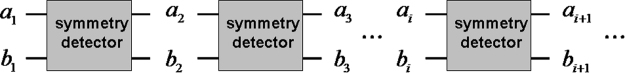
Efficient optical quantum circuit for generating two-pair by cascading symmetry detectors.

As an important application of the symmetry detectors, we now present a scalable scheme for generating the two-pair with the general four-photon entangled states. For this purpose, we construct a quantum circuit diagram by cascading these symmetry detectors, as shown in Fig. 2, where the input/output modes correspond to the signal photons. In each symmetry detector, we here simplify the initial model straightforwardly by discarding the result of the asymmetric state.

Then, after the *i* th cascading, one can obtain the symmetric state

9 $$|{{\rm{\Phi }}}_{x}^{i}\rangle ={N}_{i}\mathrm{(|2},0;0,2\rangle +\mathrm{|0},2;2,0\rangle -{c}_{i}\mathrm{|1},1;1,1\rangle ),$$

where *c*_*i*_ = 2/(1 + *c*_*i*−1_), ${N}_{i}^{2}=1/\mathrm{(2}+{c}_{i}^{2})$. The total success probability reads

10 $$P=\prod _{i}\,{P}_{i},\quad {P}_{i}=1/\mathrm{\{1}+{\mathrm{(1}-{c}_{i-1})}^{2}/\mathrm{[2}+{\mathrm{(1}+{c}_{i-1})}^{2}\mathrm{]\}.}$$

As a result, it is not difficult to find that such a cascading symmetry-detector is capable of generating two-pair from the mentioned four-photon entangled states.

**Figure 3:**
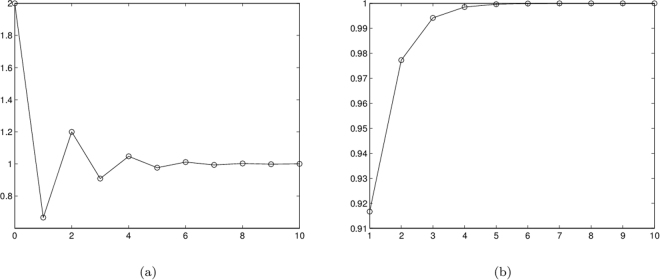
Two relationships of the coefficients *c*_*i*_ and probabilities *P*_*i*_ versus the number of iterations *i* (*i* = 1, 2, $\cdots$, 10). By a few iterations, (**a**) *c*_*i*_ gets close to 1 and (**b**) *P*_*i*_ may quickly be close to 1.

Clearly, we here take *c* = 2 for example. We calculate the iterative coefficient *c*_*i*_ and the probability *P*_*i*_ and plot the relationships of the correlation coefficients and the success probabilities versus the number of iterations (10 times), as shown in Fig. 3. The result shows that with a few iterations the correlation coefficient approaches 1 and the success probability gets close to 1. Furthermore, since we take only those events into account that yield the required results via postselection, the present scheme of the two-pair generation is near deterministic.

### Symmetry detector for the higher-order emissions

So far, we have addressed the symmetry detector and its interesting application, involving the second-order emission of the SPDC process. In order to enable us to explore multiphoton entanglement with a large number of photons from SPDC source, we now describe a method to generalize the symmetry detector from the second-order to the higher-order emissions of the SPDC source.

For the higher-order emissions, consider an *n*-pair multiphoton entangled state $|{\psi }_{n}^{-}\rangle$. When the photons passing through the 50:50 BS, the transformation between the incoming modes (*a*_1_ and *b*_1_) and the outgoing modes (*a*_2_ and *b*_2_) is

11 $${({a}_{{1}_{H}}^{\dagger }{b}_{{1}_{V}}^{\dagger }-{a}_{{1}_{V}}^{\dagger }{b}_{{1}_{H}}^{\dagger })}^{n}\to {({a}_{{2}_{H}}^{\dagger }{b}_{{2}_{V}}^{\dagger }-{a}_{{2}_{V}}^{\dagger }{b}_{{2}_{H}}^{\dagger })}^{n}\mathrm{.}$$

Then, as the multiphoton interference effect at the symmetric BS, the input *n*-pair entangled state $|{\psi }_{n}^{-}\rangle$ will be transformed into

12 $$\begin{array}{rcl}|{{\rm{\Phi }}}_{{\rm{BS}}}^{n}\rangle  & = & \frac{1}{\sqrt{n+1}}\frac{1}{n!}{({a}_{{2}_{H}}^{\dagger }{b}_{{2}_{V}}^{\dagger }-{a}_{{2}_{V}}^{\dagger }{b}_{{2}_{H}}^{\dagger })}^{n}\mathrm{|0}\rangle \\  & = & \frac{1}{\sqrt{n+1}}\sum _{k=0}^{n}\,{(-\mathrm{1)}}^{k}|n-k{\rangle }_{{a}_{{2}_{H}}}|k{\rangle }_{{a}_{{2}_{V}}}|k{\rangle }_{{b}_{{2}_{H}}}|n-k{\rangle }_{{b}_{{2}_{V}}}\mathrm{.}\end{array}$$

This result implies that when the photons passed through a symmetric BS the *n*-pair entangled state remains unchanged.

In the process of the nonlinear interactions, for clearer statement, we here rewrite the Kerr phase shifts *θ*/3 and 2*θ*/3 in spatial modes *a*_2_ and *b*_2_, and the original phase shift −5*θ* is accordingly replaced by −*θ*. On the basis of the methods of exploring multiphoton entanglement via weak nonlinearities^44,45^, for arbitrary *m*-pair, 1 ≤ *m* ≤ *n*, the total phase shift in the probe mode is (*m* − 1)*θ*. Then after an *X* homodyne measurement, one may obtain the *n*-pair with the value *x* < *α*{cos[(*n* − 1)*θ*] + cos[(*n* − 2)*θ*]}, (*n* − 1)-pair with *α*{cos[(*n* − 1)*θ*] + cos[(*n* − 2)*θ*]} < *x* < *α*{cos[(*n* − 2)*θ*] + cos[(*n* − 3)*θ*]}, $\cdots$, *m*-pair with the value *α*{cos(*mθ*) + cos[(*m* − 1)*θ*]} < *x* < *α*{cos[(*m* − 1)*θ*] + cos[(*m* − 2)*θ*]}, $\cdots$, two-pair with *α*[cos *θ* + cos 2*θ*] < *x* < *α*[1 + cos *θ*] or one-pair (the singlet state) with *x* > *α*[1 + cos *θ*]. Obviously, as a particular case of the *n*-pair emissions, a one-pair emission is simple but instructive. Since these multipair structures are robust against losing of photons they are maybe contribute to explore multiphoton entanglement from microscopic to macroscopic systems.

## Discussion

By now, we have concentrated on the means to explore symmetry detectors for twin-beam multiphoton entanglement. A realistic SPDC source with the higher-order emissions, however, inevitably emits one-pair, two-pair or *n*-pair entangled photons, spontaneously. A surprising result of the present symmetry detector is that the number of the pairs emitted from the SPDC source can be determined exactly and then be collected. Indeed, after the *X* homodyne measurement on the probe beam one can immediately infer the number of the pairs by means of the value of measurement. Also, the signal photons are specifically projected onto a particular multipair entangled state.

In recent years, one of the most intriguing developments of quantum theory, both theoretical and experimental, is optics-based quantum information processing^3,5,6,48–50^. However, many fundamental challenges remain for practical application, for example, how to obtain (approximately) *π*-radian phase shifts with cross-Kerr nonlinearities. In 2003, theoretically, Hofmann *et al*.^51^ showed that a nonlinear phase shift of *π* can be obtained by using a single two-level atom in a one-sided cavity. However, it exists a challenge to experimental realization due to additional reflections of mismatched pump light. A recent study^52^ showed that it is still unsatisfactory for creating high-fidelity *π*-radian conditional phase shifts by the cross-Kerr effect in optical fiber. In view of this fact, we here only take weak nonlinearities into account, i.e. *θ* ≤ 10^−2^. More concretely, if we take *α* = 2.0 × 10^6^ and *θ* = 2.0 × 10^−3^, then the error probability of our symmetry detector is $\varepsilon ={\rm{erfc}}\,({x}_{d}/2\sqrt{2})/2\simeq 3\times {10}^{-5}$, where *x*_*d*_ is the distance between two peaks of Gaussian curves. Furthermore, it is exactly the maximal value of the error probabilities in the process of determining the multipair emissions. Therefore, it is in this sense that the present scheme can be realized in a nearly deterministic manner.

In conclusion, we explore an efficient symmetry detector for detecting the twin-beam multiphoton entanglement based on BS and weak nonlinearities. Especially, as a typical application we suggest a scalable scheme of two-pair generation with a class of four-photon entangled states by cascading these symmetry detectors. Note that such two-pair entangled states may be very useful for multiphoton quantum information processing in the future. In the present architectures, there are several remarkable advantages. First, for the higher-order emissions, it is capable of determining the number of the pairs emitted indistinguishably from the SPDC source. Second, since we here only use a symmetric BS and two small Kerr nonlinearities, our symmetry detector is simple and novel. At last, it is possible to extend our means to general circuits constructed from linear elements, SPDC sources, and detectors. We hope that our scheme will stimulate investigations on the applications of higher-order emissions from the SPDC source.
